# 326. Impact of PCV13 introduction on the evolution of multidrug resistance (MDR) in S. pneumoniae causing invasive pneumococcal disease (IPD) in Argentina

**DOI:** 10.1093/ofid/ofaf695.115

**Published:** 2026-01-11

**Authors:** Paula Gagetti, Jonathan C Zintgraff, Nahuel Sanchez Eluchans, Paulina Marchetti, Mariano Echegorry, Claudia Lara, Alejandra Corso

**Affiliations:** INEI-ANLIS DR CARLOS G MALBRAN, CABA, Ciudad Autonoma de Buenos Aires, Argentina; INEI-ANLIS DR CARLOS G MALBRAN, CABA, Ciudad Autonoma de Buenos Aires, Argentina; INEI-ANLIS DR CARLOS G MALBRAN, CABA, Ciudad Autonoma de Buenos Aires, Argentina; INEI-ANLIS DR CARLOS G MALBRAN, CABA, Ciudad Autonoma de Buenos Aires, Argentina; INEI-ANLIS DR CARLOS G MALBRAN, CABA, Ciudad Autonoma de Buenos Aires, Argentina; Instituto Nacional de Enfermedades Infecciosas ANLIS "Dr Carlos G Malbran", Ciudad Autónoma de Buenos Aires, Ciudad Autonoma de Buenos Aires, Argentina; Instituto de Enfermedades Infecciosas ANLIS "Dr Carlos G Mabrán", Buenos Aires, Buenos Aires, Argentina

## Abstract

**Background:**

*S. pneumoniae* (Spn) is a major human pathogen causing infections in children and elderly. The National Surveillance of Spn serotypes and antimicrobial resistance for IPD is conducted in Argentina since 1993. The objective was to evaluate the evolution of multiple resistance (MDR, resistance to ≥3 families of antibiotics) in < 6 years after the introduction of PCV13 to the national vaccination schedule in 2012.

**Figure 1.**
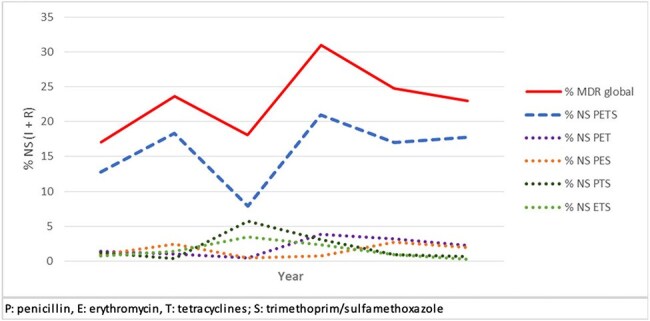
Biannual evolution of MDR in S. pneumoniae causing IPD and MDR phenotypes, n: 1582

**Methods:**

Between 2013 and 2024, 1582 IPD isolates from < 6 years were submitted from hospitals of all 24 jurisdictions, 344 (21.7%) of them were MDR. Serotyping was performed by Quellung and MICs by agar dilution (CLSI 2025). A subset of serotype 19A and 24F/A/B was studied by WGS (Illumina).

**Results:**

The 344 MDR were differentiated into 33 serotypes, of which 24F/A/B (52%), 19A (17%), 14 (6%) and 15B/C (5%) accounted 80%. MDR and resistance phenotypes showing fluctuations with an upward trend (Figure 1). MDR increased from 17% in 2013-14 to 23% in 2023-24 (p=0.058), mainly associated with NS-PETS (penicillin, erythromycin, tetracycline, and SXT). Increases in MDR were associated with decreases in serotypes 14 (9.9% vs. 4.2%) and 6B (7% vs. 0%) and increases in 24F/A/B (50.7% vs. 56.3%) and 19A (9.9% vs. 16.9%). Serotype 19A associated to CC172-GPSC5 PEN MIC_50/90_ 0.12/0.5 mg/L, non-MDR in 2013-14, was replaced by CC320-GPSC1 PEN MIC_50/90_ 2/4 mg/L and MDR in 2023-24. Serotypes 24F/A/B NS-PETS MDR were associated with CC230-GPSC10 throughout all the period.

**Conclusion:**

The evolution of MDR in Spn in Argentina post-PCV13 is associated with the increase in non-vaccine MDR serotypes (24F/A/B) and the clonal replacement of vaccine serotype 19A, which continues to be prevalent in the post-PCV13 era, associated with MDR in recent years. National surveillance of IPD is relevant for assessing changes in epidemiology, the impact of vaccines, and defining empirical treatments.

**Disclosures:**

All Authors: No reported disclosures

